# Supplementary material for: Are carbon emissions trading and green financial instruments synergistic? -Comprehensive quantitative research based on content analysis
Source: PLoS One. 2024 Mar 7;19(3):e0298601. doi: 10.1371/journal.pone.0298601 (PMC10919601; doi:10.1371/journal.pone.0298601)
Supplement: S1 Data — (ZIP) [file pone.0298601.s001.zip › Data/地级市Z_F工作报告.zip!/╣π╢1⁄2/╟σ╘╢╩╨/└┤╫╘╣1⁄2╓┌║┼í╛┬φ┐╦╩2╛▌═°í┐_2011.docx]

各位代表：

我代表市人民政府向大会报告政府工作，请予审议，并请政协各位委员和其他列席人员提出意见。

一、“十一五”时期工作回顾

“十一五”时期是充满压力和挑战的五年，也是我市经济社会实现跨越式发展的五年。面对国际金融危机的冲击和雨雪冰冻等自然灾害影响，市政府在省委、省政府和市委的正确领导下，在市人大、市政协的监督支持下，团结带领全市人民，深入贯彻落实科学发展观，大力推进“三促进一保持”，坚持扩总量与促转变并举，坚持惠民生与保稳定并重，全力推动经济社会发展，2010年各项目标顺利实现，“十一五”规划任务超额完成。

（一）全力抓提速扩总量，经济发展实现历史性跨越

经济实力迈上新台阶。主要经济指标增速连续五年居全省首位，总量在全省排位大幅提升。2010年，全市生产总值突破千亿元，达1112.5亿元，比上年增长20.8%，比2005年增长1.9倍，年均增长24%，总量排全省第12位；人均生产总值2.89万元，比上年增长18.9%，比2005年增长1.7倍，年均增长22.1%；来源于清远的财政总收入170.7亿元，比上年增长47.1%，是2005年的5.7倍，年均增长41.83%；地方财政一般预算收入72.8亿元，比上年增长45.5%，是2005年的5.5倍，年均增长40.6%，总量居粤东西北各市第一位。在2010年中国社科院发布的《城市竞争力蓝皮书》中，我市被列为连续5年稳定增长的十个城市之一，综合增长竞争力排名第5位，是粤港澳地区唯一入选城市。

工业经济发展迅猛。2010年，全市规模以上工业增加值611.2亿元，比上年增长34.6%，比2005年增长5.5倍，年均增长45.6%；工业总产值突破3000亿元，比上年增长52.2%，比2005年增长5.9倍，年均增长47.3%，工业经济总量列全省第9位。工业经济效益大幅提升，2010年全市规模以上工业企业实现利润51.6亿元，比上年增长77.2%，比2005年增长18.4倍。

农业产业化水平明显提升。2010年农业增加值120亿元，比上年增长7.2%，五年年均增长5.4%。着力完善农业基础设施，加快农业产业基地建设，积极引进和培育农业龙头企业，市级以上农业龙头企业增至100家，具有一定规模的农业产业化组织增至196个，带动35.9万农户年均增收3966元；无公害农产品产地认定企业发展到144家，共有无公害农产品认证195个、有机食品认证产品9个、绿色食品认证产品25个。外向型农业加快发展，备案（注册）农产品出口基地发展到58家。

第三产业快速发展。积极推进金融、物流、房地产等现代服务业发展，成立了6家小额贷款公司，融资性担保机构发展到32家、注册资本金17.85亿元；2010年金融机构本外币存、贷款余额分别为995.36亿元、520.62亿元，分别比2005年增长1.5倍和1.9倍；保险费收入19.62亿元，比2005年增长1.83倍。世界500强零售企业沃尔玛落户英德市，清远义乌商贸城、中汽南方国际汽车城等专业市场建设顺利推进。房地产业异军突起，2010年全市商品房销售额135.3亿元，比上年增长9.13%，是2005年的15.2倍。旅游业竞争力不断提升，2010年接待海内外游客2169万人次，旅游总收入108.4亿元，分别比上年增长15.3%和44.4%，分别是2005年的2.16倍和3.3倍；拥有4A级景区10个，名列全省地级市之首；连州地下河申报5A级景区已通过专家评审。2010年第三产业增加值318.2亿元，比上年增长10.3%，五年年均增长16.4%。

经济发展内生动力增强。大力实施扩内需战略，积极应对国际金融危机冲击，投资需求和消费需求有效扩大，对经济增长的拉动作用不断增强。五年累计完成固定资产投资3357.8亿元，年均增长34.5%，是“十五”时期的5.6倍，其中2010年达996.9亿元，比上年增长18.5%，规模升至全省第5位；全社会消费品零售总额370.5亿元，比上年增长22.4%，比2005年增长1.9倍，年均增长23.4%。

（二）大力推进结构调整，经济发展方式加快转变

自主创新能力明显提升。积极推进产业基地和创新平台建设，五年共组建省级工程技术研究开发中心4家、市级工程技术研究开发中心6家、省级农业科技创新中心2家，创建省火炬计划特色产业基地3个、专业镇创新服务平台8个。2010年全市研发经费支出比上年增长25%，荣获“2010中国最具创新力城市”称号。组建了“广东省再生金属综合利用产学研创新联盟”，全市62家企业与56所高校、科研院所开展了产学研合作，63名教授（博士）作为科技特派员进驻49家企业。五年专利申请量1291件，专利授权量920件，分别是2005年的2.78倍和4.95倍。其中2010年专利申请量465件、授权量416件，分别比上年增长92.2%和1.77倍，增速均居全省首位。

产业结构进一步优化。积极推进产业结构调整，加快传统产业改造提升，五年共投入更新改造资金428.3亿元，是“十五”时期的4.2倍。积极推动产业集聚发展，启动了省市共建（清远）再生有色金属先进制造业基地建设，清城有色金属再生产业集群、佛冈龙山空调制冷配件产业集群被认定为省产业集群升级示范区，有色金属加工、再生资源产业集群初具雏形。加快先进制造业和高新技术产业发展，国家高新技术企业增至29家，经省科技厅认定的高新技术产品达46个。2010年高新技术企业实现工业总产值62.8亿元，销售收入57.06亿元；高技术制造业增加值占工业增加值的5.8%，比上年增长34.9%，已成为经济发展新的增长点。三次产业结构由2005年的22.1：39.3：38.6调整为2010年的10.8：60.6：28.6，工业对经济增长的主导作用进一步增强。

品牌创建成果丰硕。大力实施质量兴市、品牌带动战略，参与制定国家、行业、地方标准14项，国家水泥及制品质量监督检测中心落户我市；“新亚”牌和“光”牌商标被认定为中国驰名商标，实现了驰名商标零的突破；清远鸡、东陂腊味、英石等12个地方特色产品获得国家地理标志产品保护，数量居全省之首；拥有特色农产品地理标志证明商标3件；36个产品获国家、省名牌产品称号；36件商标被认定为广东省著名商标，全市名牌产品、著名商标已增至89个（件）。

节能减排目标顺利完成。强化节能预警调控，加快淘汰落后产能，五年共淘汰小水泥、小钢铁企业230多家，涉及产值100多亿元。大力推广余热发电技术，共建成余热发电机组12台，总装机容量15.25万千瓦。加强污染减排设施建设，强化重点流域及重点排污企业监管，燃煤火电机组全部安装了脱硫设施；各县城和中心镇全部建成污水处理厂，五年共新（扩）建15间污水处理厂，新增污水日处理能力29.5万吨，是2005年的6.9倍。单位GDP能耗、二氧化硫和化学需氧量排放量顺利完成“十一五”目标。

节地工作取得新成效。优化土地资源配置，深挖用地潜力，推进土地节约集约使用，单位建设用地GDP产出从2005年的每亩2.56万元提高到2010年的7.26万元，建设用地利用效率提高1.84倍。积极推进开发补充耕地工作，三年累计开发补充耕地21.53万亩，连续两年排全省第二位。全面开展“三旧”改造工作，近三年共投入改造资金66.9亿元，完成改造项目90个，涉及面积1.39万亩，节地率43%。

（三）狠抓“双转移”和“双到”工作，区域发展协调性增强

“双转移”工作深入推进。狠抓工业园区建设，佛山（清远）、佛山禅城（清新）产业转移园创建成为省级产业转移园。华侨工业园、民族工业园基础配套不断完善，吸引力有效增强。世界500强企业法国液化空气集团落户佛山（清远）产业转移园。2010年，四大园区共引进项目90个，投资总额129.1亿元，实现产值114.2亿元。创新“双转移”模式，顺德（英德）区域合作产业园正式启动，探索出了区域经济合作的新路子。以园区为载体，大力承接国际国内尤其是珠三角产业转移，五年共引进项目2933个，计划总投资3834.99亿元，累计实际投入998.06亿元。切实抓好农村劳动力实用技能培训，促进劳动力转移就业。五年累计培训农村劳动力14.6万人，转移农村劳动力61.61万人次，其中就地就近就业35.56万人。

扶贫开发成效显著。积极实施农村安居工程，加大扶贫开发力度，贫困地区生产生活条件进一步改善。积极推进扶贫开发“规划到户、责任到人”工作，共落实“双到”资金5.8亿元，已投入2.45亿元；共扶持发展集体经济项目1000个，235个省级贫困村集体经济收入超过3万元，16万贫困人口年人均收入超过2500元，清城区提前一年实现脱贫目标。积极开展“扶贫济困日”活动，广泛动员社会力量参与扶贫，共募集善款2.3亿元。

县域经济实力大幅提升。坚持分类指导，积极推进主体功能区规划试点工作，因地制宜发展特色产业，北部四县（市）经济发展速度加快，五年年均增速均达到两位数。南部和中部地区综合实力不断增强，2010年《广东县域经济综合发展力研究报告》显示，佛冈县、英德市、清新县经济综合发展力分别在全省67个县（市）中位列第7、第10和第11位，包揽全省山区市县域经济综合发展力前三名。

民族地区发展加速。认真贯彻落实民族政策法规，出台了《关于用办特区的理念加快少数民族地区发展的实施意见》，市财政五年共投入两个民族自治县扶持资金8602万元，是“十五”时期的9.4倍。创办了民族工业园，突出抓好民族地区基础设施建设，有力促进了民族地区经济社会发展。2010年，连山、连南两个民族自治县生产总值分别增长20.1%、20.2%，分别比2005年提高8.9和7.9个百分点。两个自治县公路通车里程1962.1公里，公路密度76.3公里/百平方公里；实现了城乡免费义务教育，小学、初中入学率超过99%和97%；合作医疗覆盖率超过98%。

（四）加大城乡建设力度，城镇化水平进一步提升

基础设施日益完善。以交通建设为重点，加快城乡基础设施建设，城乡面貌焕然一新。五年共完成基础设施建设投资800多亿元，其中交通设施投资370多亿元。完成了清连、广清和京港澳高速公路清远段、武广高铁清远段、北江大桥姐妹桥、凤城大桥等重大交通项目建设，实现了县（市、区）到镇通等级公路和镇到建制村公路路面硬化。全市公路通车里程1.81万公里，五年新增7378公里，其中高速公路240公里；公路密度从2005年的每百平方公里56.45公里提高到95.96公里。五年共投入电网建设资金71.97亿元，投产110千伏及以上线路1460.98公里，投产110千伏及以上变电容量558.6万千伏安，分别是“十五”时期的3.73倍、4.45倍和4.81倍。共解决117.4万人的饮水安全问题，提前三年完成省下达任务；完成堤防建设和加固245.35公里、病险水库除险加固工程102座、省人大议案机电排灌工程107宗，五年共投入水利建设资金58.06亿元，比“十五”时期增长1.52倍。信息化建设成效显著，数字城市建设顺利推进，互联网用户153.6万户，移动电话用户277.2万户，分别比2005年增长7倍和2.43倍。

城镇化进程明显加快。启动“十个一批”城乡重点工程建设，完成了城西大道扩建、连江西路、沿江路、“西出口”等一批市政交通项目，以及清远站站前广场、笔架广场、凤城文化广场和中山公园、汝珍公园、秀丽公园等一批广场、公园改（扩）建工程，市区首座人行天桥建成通行，各县城中轴线、水轴线全面完成，城市配套功能不断完善，综合服务能力显著提升。城市规划和管理机制进一步完善，完成了《清远市城市总体规划（2010—2020）》及“一江两岸”城市设计等专项规划的编制，理顺了市、区两级城市管理体制。市区建成区面积56.12平方公里，绿地面积1975.4公顷，绿化覆盖率40.12%，人均公园绿地面积11.27平方米，分别是2005年的1.42倍、1.66倍、1.19倍和1.97倍。

宜居城乡建设加快推进。实施城乡清洁工程，大力开展“三边”整治，积极推进新农村建设，“八十村示范、千村整治”工程、5个新农村建设示范片和佛冈县共建新农村先行试验区取得新成效，城乡美化、绿化、亮化水平大幅提升，人居环境明显改善。积极推进生态建设，五年共营造生物防火林带2220.4公里、面积2548.3公顷；清城区、英德市、清新县和连山县创建成为省林业生态县，全市森林覆盖率达67.8%。城市生活污水处理率61.52%，生活垃圾无害化处理率48.16%，分别比2005年提高2.67和23个百分点。加强水资源和环境保护，主要江河水质保持良好，饮用水源水质达标率100%；城市空气质量达到国家二级标准，城市声环境保持稳定。

（五）积极推进改革开放，市场经济体系不断完善

重点领域改革扎实推进。市县两级政府机构改革基本完成，大部门体制改革顺利启动。完成了第三、第四轮行政审批制度改革，建立了“一网式”和并联式审批系统，成为审批事项最少的山区市。建立和完善市与区利益共同体体制机制。简政强镇事权改革加快推进，事业单位分类改革有序开展。扎实推进行政区域合并工作，乡镇、村委会撤并率分别达44%和30%。市属政府投资非经营性项目全面实行代建制。金融机构改革进一步深化，市农信社统一法人工作进展顺利，城信社退出市场工作全面完成。污水、垃圾处理收费改革取得阶段性成果。教育、卫生、文化体制改革深入推进。集体林权制度主体改革任务基本完成，华侨农场改革发展取得重大进展。

多种经济成分共同发展。加快国有企业改革，五年共完成128户国有企业的改革改制，国有企业发展活力进一步增强，国有经济不断发展壮大。市属监管企业国有资产总额发展到97.15亿元，是2005年的22倍；所有者权益21.1亿元，是2005年的10.2倍，国有资本保值增值率超过9倍。认真落实加快民营经济发展各项政策，切实抓好中小企业帮扶工作，民营经济发展不断加快。2010年全市实有个体工商户、私营企业分别为9.12万户、9002户，分别比2005年增长33.73%和1.53倍；民营经济实现增加值495.2亿元，占全市比重的44.5%，是2005年的3.2倍。新亚电缆、远光电缆及忠华棉纺等3家企业入围全省民营企业百强。

外经贸发展步伐加快。五年共引进外资项目394个；合同外资金额26.15亿美元，实际利用外资21.56亿美元，分别比“十五”时期增长24%和2.2倍。积极开展对外贸易，促进加工贸易转型升级。2010年外贸出口总额19.3亿美元，比上年增长36.7%，是2005年的2.1倍，年均增长15.7%；外贸出口结构进一步优化，一般贸易出口4.4亿美元，比上年增长33.2%，是2005年的1.2倍；机电产品和高新技术产品出口分别比上年增长28.6%和12.8%，比2005年提高2.03倍和2.76倍。

（六）切实加强和改善民生，社会各项事业全面进步

人民生活质量稳步提升。编制实施基本公共服务均等化规划纲要，五年共投入改善民生资金211.3亿元，2010年财政民生投入占一般预算支出比重达47.53%。城乡居民收入稳步提高，2010年市区城镇居民人均可支配收入1.58万元，农村居民人均纯收入6386元，分别比2005年增长69.1%和60%。企业职工最低工资水平比2005年提高60.9%。发行了清远市民卡。认真落实促进就业各项政策，积极帮扶大学生、农民工等群体就业，五年新增城镇就业30.4万人，城镇登记失业率控制在3.5%以内，零就业家庭保持动态为零。企业人文关怀得到加强，劳动关系总体保持和谐稳定。

社会保障体系进一步完善。积极拓展城镇居民基本医疗保险、农村合作医疗、农村养老保险和失地农民养老保险覆盖面，社会保障体系基本覆盖城乡。2010年全市参加新型农村养老保险12.48万人，比上年增长3.2倍；新农合参合率98.38%；参加城镇基本医疗保险84.62万人，比上年增长8.02%，比2005年增长3.87倍；参加失地农民养老保险1.43万人。社保资金累计结余32.1亿元，是2005年的2.8倍。符合低保条件的城乡居民实现应保尽保。社会救助体系进一步完善，社会福利和红十字等慈善事业持续发展。出台政策稳定物价水平，首次向低收入群体发放临时价格补贴。积极推进保障性住房建设，共建成保障性住房1263套。

文化建设加快推进。社会主义核心价值体系建设扎实推进，精神文明创建活动深入开展。加强公共文化服务体系建设，博物馆、图书馆等文化基础设施不断完善，农村电影放映工作走在全省前列，农家书屋建设提前5年实现行政村全覆盖。深入开展文化品牌创建活动，中国漂流之乡、中国温泉之乡影响不断扩大，连州国际摄影年展、英德英石文化节、阳山四驱越野车节、连山壮家戏水节、连南瑶族文化艺术节知名度不断提高。加强文化遗产的保护和利用。福地、英石和壮瑶等文化产业不断发展。新华书店和电影公司成功转企改制，重新组建了市民族歌舞团。

教育优先发展地位得到巩固。全面实施城乡免费义务教育，提前一年实现“普高”目标，新增普通高中学位2.41万个、中职（含技工）学位4.55万个；2010年高中阶段教育毛入学率93.08%，比2005年提高42.08个百分点；清远职业技术学院办学条件和内涵建设不断提升，全市高等教育在校生1.21万人，毛入学率28.5%。加快发展学前教育、特殊教育，积极引进民间资本办学，民办教育在校生9.7万人，比2005年增长6倍。教育创强成绩显著，清城区创建为非珠三角地区第一个省教育强区，佛冈县创建为全省第一个省教育强县，累计创建省教育强镇18个、国家级示范性普通高中10所。全面实施教师绩效工资改革，教师工资福利待遇实现“两相当”，全面解决代课人员“代转公”问题。

卫生、体育等社会事业取得新成效。加快卫生事业发展，基层医疗卫生服务体系建设不断推进，医疗条件明显改善，疾病预防控制、妇幼保健服务、农村（社区）卫生服务上新水平。扎实推进卫生资源优化整合，市人民医院已完成整体搬迁并正常运作。基本药物制度全面启动，中医药事业不断进步。突发公共卫生事件应急机制逐步健全。体育事业蓬勃发展，成功举办第四届市运会，全面完成农村体育健身工程建设，群众性体育运动广泛开展。完成省下达的人口计划各项指标任务，人口自然增长率控制在7‰左右，低生育水平继续保持稳定。妇女儿童、残疾人、侨务、统计、人防、气象、档案、方志、对台等工作取得新成绩。

民主法治和政府自身建设进程加快。自觉接受市人大及其常委会的监督，积极开展民主协商工作，开展人大代表、政协委员约见市长活动，五年共办理人大代表建议294件、政协提案602件，人大代表满意率达100%，政协委员满意率达96%以上。密切政府与各民主党派、工商联、人民团体、无党派代表人士的联系，科学民主依法决策机制不断完善。加强机关作风建设，近两年共对全市737个机关单位进行了433次明查暗访，制作机关作风暗访专题片13部；共受理行政效能投诉357件，办结率达100%。全面完成“五五普法”，法治城市、法治县（市、区）创建活动不断深入。监察、审计工作扎实推进，政务、村务和办事制度公开不断加强，惩治和预防腐败体系进一步完善。

社会大局保持稳定。着力做好信访工作，认真落实领导接访和包案制度，全市信访形势总体平稳。加大社会治安治理力度，县镇村综治信访维稳中心全面建成，社会政治和社会治安保持稳定。深入开展打私、打假工作，狠抓产品质量和食品药品安全专项整治，打击传销工作取得阶段性成果，市场秩序不断规范。应急管理体系进一步完善，预防和应对突发事件能力有效提升。切实抓好安全生产，有效防范了重特大安全事故的发生，2010年各类事故起数和死亡人数比2005年分别下降61.2%和40.7%。民族、宗教关系和睦和谐。国防建设和民兵预备役工作得到加强，军民共建、双拥优抚安置深入推进，军政军民更加团结。

各位代表，“十一五”时期是我市经济实力提升最快、城乡面貌变化最大、社会各项事业进步最为显著、人民群众得到实惠最多的五年，是具有里程碑意义的五年。这一业绩的取得，是我们坚持邓小平理论和“三个代表”重要思想、全面贯彻落实科学发展观的结果，是省委、省政府和市委正确领导的结果，是各级人民代表大会及其常务委员会和人民政协监督支持的结果，是全市人民同心同德、奋发图强、开拓创新的结果，是港澳台同胞、海外侨胞和社会各界关心支持的结果，是兄弟地区尤其是挂扶单位大力帮助的结果。在此，我代表市人民政府，向全市广大工人、农民、知识分子、干部职工、驻清人民解放军、武警消防官兵、人民警察和各民主党派、工商联、人民团体、社会各界人士表示崇高的敬意！向长期关心和支持清远建设的兄弟地区、香港和澳门特别行政区同胞、台湾同胞、海外侨胞和国际友人，表示衷心的感谢！

各位代表，通过“十一五”时期的成功实践，我们进一步提高了驾驭全局和解决复杂问题的能力，探索和积累了推动经济社会发展的经验。第一，坚持以科学发展观统揽全局，是推动清远跨越发展的思想指南。深刻领会科学发展观的实质内涵，转变粗放型发展路径和理念，注重速度与质量的统一，努力寻求破解发展难题的新途径，推动了经济与社会的又好又快发展。第二，坚持改革创新，是推动清远跨越发展的强大动力。以解放思想、创新观念为先导，深入推进重点领域和关键环节改革攻坚，不断创新政策、体制机制，积极探索符合清远实际的发展路子，最大限度释放了发展活力，为加快发展注入了强大动力。第三，坚持统筹城乡区域发展，是推动清远跨越发展的重要抓手。坚持全面谋划、统筹协调、分类指导，深入推进“双转移”和“双到”扶贫开发，抓好“三圈四带”产业布局，积极开展主体功能区规划试点工作，促进了城乡和区域的协调发展。第四，坚持调结构促转变，是推动清远跨越发展的必由之路。紧紧把握国际金融危机带来的倒逼压力和转型契机，积极推进产业和经济结构调整，提高自主创新能力，加快发展高端产业，提高产业发展水平，有效提升了经济发展质量。第五，坚持以人为本、改善民生，是推动清远跨越发展的根本宗旨。破除“重物轻人”的观念，把解决民生问题作为发展的前提与归宿，积极推进教育、文化、卫生等各项事业发展，不断提高人民群众物质文化和社会保障水平，扎扎实实为群众谋福祉。第六，坚持提高政府执行力，是推动清远跨越发展的重要保障。积极推进行政审批制度改革，狠抓公务员队伍建设，不断加强政府自身建设，积极推进政务公开，切实提高政府依法行政和高效服务水平。大力倡导真抓实干的作风，完善责任落实机制，狠抓督促检查，推动了各项工作的有效开展。

回顾“十一五”时期的工作，我们也清醒认识到，我市经济社会发展还存在不少困难和问题：一是经济发展方式总体粗放，产业层次不高，自主创新能力弱，高新技术产业比重偏低，服务业发展相对滞后，产业竞争力不强；二是区域和城乡发展不平衡问题仍然突出，完成扶贫开发目标、加快北部地区发展任务艰巨；三是城镇化水平较低，城市辐射带动作用不强；四是资金、土地等要素供应不足问题日益突出，缺乏大项目带动，发展后劲受到制约；五是文化、卫生等社会事业发展还需加大力度，推进基本公共服务均等化任重道远。这些困难和问题，我们将在“十二五”时期努力加以克服和解决。

二、“十二五”时期的奋斗目标和主要任务

“十二五”时期是我市深化改革开放、加快转变经济发展方式的攻坚期，也是我市建设全面小康社会、推动经济社会实现更大跨越的关键期。我们要准确把握形势，积极应对各种挑战，开拓创新，扎实工作，努力开创发展的新局面。

根据党的十七届五中全会、省委十届八次全会精神和市委关于“十二五”规划的建议，市政府组织编制了《清远市国民经济和社会发展第十二个五年规划纲要（草案）》。《规划纲要（草案）》及其说明已印发大会。《规划纲要（草案）》提出，“十二五”时期工作的总体要求是：高举中国特色社会主义伟大旗帜，以邓小平理论和“三个代表”重要思想为指导，深入贯彻落实科学发展观，以科学发展为主题，以加快转变经济发展方式为主线，以“加快四化进程，建设幸福清远”为核心，全面推进经济、社会、文化、政治和生态文明建设，实现经济与社会、环境的协调发展、城乡区域的统筹发展、人与自然的和谐发展，努力打造广东区域协调发展示范区、环珠三角高端产业成长新区、华南休闲宜居名城和大广州卫星城，为全面建成小康社会打下坚实基础。

“十二五”时期我市经济社会发展的主要目标是：经济实力显著增强，生产总值年均增长15%以上，人均生产总值年均增长14%以上，经济结构明显优化，城乡发展显著加快，民生质量显著提高，社会事业明显提升，生态环境明显改善。

根据上述总体要求，实现“十二五”规划目标，必须在“六个突出提升”上下功夫：

（一）突出提升基础设施建设水平。按照“建设大交通、融入大广佛、对接珠三角”的总体思路，高起点规划建设交通网络，重点围绕中心城区，以及南部、中部、北部重要工业园区、特色产业集聚带等，加强公路、铁路、航道、港口等交通基础设施建设，逐步实现与广佛都市圈一体化及周边县、市的无缝对接和快速通达。突出抓好民族地区交通、能源等基础设施建设，进一步优化民族地区发展环境。加快能源、水利等基础设施建设，实施“数字清远”工程，为经济社会发展提供强有力支撑。

（二）突出提升产业发展水平。积极承接珠三角和国际产业转移，着力发展与珠三角相配套的先进制造业和现代服务业，做大做强具有比较优势的特色产业，大力培育战略性新兴产业，以先进技术改造提升传统产业，推动产业结构升级，努力构建结构优化、技术先进、清洁安全、附加值高、资源消耗低、吸纳就业能力强的现代产业体系。加大民族工业园扶持力度，优先安排民族地区产业项目，加快发展民族医药、民族生态旅游等特色产业。加快农业产业结构调整，创新发展具有区域特色的现代农业。

（三）突出提升城镇化发展水平。以建设大都市、大城市群的理念整合南部区域，拉大中心城区主体框架，建设“湖城”及“广清一体化都市区”，加快与广佛都市圈的对接。建立开放融合型城镇体系，拓宽城市发展空间。加快推进市政基础设施建设，增强城镇综合承载能力。注重扩容提质，将城市建设与功能再造、产业发展相结合，提升城市规划与管理水平。着力推进主体功能区试点建设，促进城乡区域协调发展。创新城镇化体制机制，加快户籍制度改革，引导人口向城镇集聚。

（四）突出提升生态环保建设水平。大力推进生态城市建设，加大环境保护力度，加快“三旧”改造和节约集约用地工作。推进再生资源体系建设，积极发展循环经济和低碳经济，逐步形成节约资源的生产方式和健康文明的消费模式，提高生态文明水平，努力建设资源节约型、环境友好型社会，全面提高可持续发展能力，构筑美好绿色家园，带动清远绿色崛起。

（五）突出提升文化强市建设水平。把教育放在优先发展的战略位置，力争2013年创建为省教育强市，高标准普及学前到高中阶段十五年教育。构建覆盖城乡的普惠型公共文化服务体系，深化文化体制改革，充分挖掘并加快文化资源向产业资源的转化，做大做强文化产业。加强公共体育设施建设，加快体育产业发展，广泛开展全民健身活动，增强居民体质。

（六）突出提升民生保障水平。坚持以人为本，更加注重包容性增长和共享式发展，切实解决好人民群众最关心、最直接、最现实的利益问题。着力构建以民生为重点的社会体系，加快推进城乡基本公共服务均等化，增强医药卫生服务能力，建设惠及全民的社会保障体系。千方百计促进就业和再就业，切实提高居民收入，让人民群众共享经济社会发展成果，建设幸福和谐清远。

各位代表，“十二五”的宏伟蓝图已经绘就，清远发展新的航程已经开启。全市人民团结一致、奋力拼搏，就一定能开创更加美好的明天！

三、2011年工作安排

2011年是实施“十二五”规划的开局之年，也是中国共产党成立90周年，做好2011年各项工作，对于巩固应对金融危机成果、确保“十二五”开好局、起好步意义重大。今年政府工作的总体要求是：深入贯彻落实科学发展观，全面落实党的十七届五中全会、中央经济工作会议、省委十届八次全会和市委五届十二次全会精神，围绕“加快四化进程，建设幸福清远”这个核心，以加快转变经济发展方式为主线，全面对接《珠江三角洲地区改革发展规划纲要》，加快推进结构调整和自主创新，加快完善交通基础设施，提升“双转移”和“双到”工作水平，提升城镇化和环境保护水平，努力发展以改善民生为重点的社会事业，开创经济社会持续健康发展新局面，为“十二五”时期的科学发展奠定基础。

今年经济社会发展的主要预期目标是：生产总值增长18%左右，人均生产总值增长16%左右；全社会固定资产投资增长16%以上；社会消费品零售总额增长20%；外贸出口增长6%；实际利用外资增长30%；地方财政一般预算收入增长20%左右；在岗职工平均工资增长10%以上；农民人均纯收入增长10%以上；居民消费价格涨幅控制在4.3%左右；城镇登记失业率控制在3.5%以内；单位生产总值能耗下降、二氧化硫和化学需氧量削减量完成省下达目标。

要实现上述目标，必须突出抓好“八个着力”：

（一）着力扩大内需，增强经济发展动力

进一步拓展消费需求。继续巩固和提升“万村千乡市场工程”，推进农贸市场改造提升，切实做好家电、摩托车下乡和以旧换新工作，千方百计扩大农村消费市场。加快专业市场建设，积极引进国内外大型商贸企业，大力开拓文化、旅游等新兴消费热点。加大收入分配调节力度，提高城乡低收入居民收入，增强消费能力。

全力扩大和优化投资。安排市重点项目111项，计划投资315亿元。继续加大续建、在建项目推进力度，加快广清、广乐、二广高速清远段、金谷大桥和武广客运专线英德站建设，确保佛清从高速公路年内动工，推动广清城际轨道尽快开工，加快推进连佛、汕湛、汕昆高速清远段前期工作，以及北江航道改造升级。加大新型工业投资力度，严格控制“两高一资”和产能过剩行业投资，优化投资结构。加快储备一批投资规模大、带动力强的重点项目。落实支持民间投资的政策措施，引导社会投资参与重点项目建设。

加强经济运行调节。全面落实中央和省稳定物价各项政策措施，加强价格监管，严肃整治恶意炒作、哄抬物价等不法行为，保持物价稳定。认真落实“菜篮子”市长负责制，切实抓好“菜篮子”基地建设。完善补贴制度，保障低收入群众基本生活。加快保障性安居工程建设，着力解决低收入群体的住房困难。认真落实中央房地产调控政策，积极发挥土地储备作用，支持自住性住房消费，促进房地产业平稳健康发展。继续做好财税征管工作，坚持依法征管、应收尽收。

增强民营经济发展活力。认真落实促进民营经济发展的政策措施，优化民营经济发展环境。继续实施重点企业服务团制度，发挥商会、协会、行会等社会团体的沟通桥梁作用，加大中小企业帮扶力度。完善中小企业信用担保体系建设，推进政、银、企、保合作，努力缓解企业融资难、担保难问题。大力培育上市后备企业，推动优质企业上市融资。

（二）着力转变发展方式，加快产业优化升级

大力推进自主创新。加快科技创新平台建设，重点推进广东低碳技术与循环经济研究院清远分院、国家水泥及制品质量监督检测中心建设。鼓励支持企业组建研发中心，强化企业在技术创新中的主体地位。深入实施技术标准战略，加快创新型企业、高新技术企业培育，重点扶持具有自主知识产权、自主品牌的创新型企业发展。充分利用现有制造业基础，积极发展汽车零配件、机械模具及通用、专用设备制造业，促进先进制造业集聚发展。加快创新型人才引进和培养。

改造提升传统产业。大力引进和推广新技术，改造再生资源、有色金属加工、水泥、陶瓷等支柱产业。实施技术改造技术创新滚动计划，推进信息化与工业化融合。加快再生资源回收利用体系、石角再生金属产业集群升级示范区和“城市矿产”示范基地建设，打造有色金属、再生资源产业集群。鼓励传统产业龙头企业参与国际、国家、行业标准的拟（制）定。

加快发展现代服务业。立足发展大旅游，重点引进大企业，深度开发旅游资源。着力开发高端旅游产品，进一步拓展珠三角、高铁沿线、华东沿海和境外高端客源市场。大力发展面向珠三角的高端会展业，加快发展物流、电子商务、金融、保险等生产性服务业，做大做强城西大道--人民路商贸物流带。强化信息技术在旅游、商贸等服务业的推广应用，促进现代服务业与先进制造业互动发展。

进一步优化外贸结构。继续做强一般贸易，提升加工贸易水平。重点支持高新技术产品、自主产品、名牌产品出口。扩大先进技术、关键设备和零部件进口。推动外商投资、加工贸易企业扩大内销。加快口岸建设，不断优化通关环境，在继续巩固欧美、日本等传统市场的同时，积极开拓东盟等新兴国家贸易市场，进一步加强与港澳台的经贸合作，提高对外开放的广度和深度。

着力抓好专业镇建设。以园区为载体，以产业为支撑，以品牌建设为抓手，加快推进清城区石角镇有色金属加工专业镇建设，促进9个省级技术创新专业镇的产业集聚和创新升级，使之成为主导产业各具特色、带动能力较强的专业镇，并通过完善配套服务功能，打造城乡统筹发展的示范镇。

（三）着力深化改革，提高地区综合竞争力

深化行政体制改革。继续推进富县强镇和简政强镇事权改革，加大简政放权工作力度，依法下放经济社会管理权限，切实强化县级政府行政管理职能。进一步推进“一网式”审批和并联审批，提高审批效率。认真做好清城区大部制改革试点工作，积极推进事业单位分类改革。

积极稳妥推进重点领域改革。推进金融改革创新，加快组建村镇银行，深化农村信用社改革，稳健发展小额贷款公司，规范发展融资性担保机构，鼓励更多股份制商业银行在我市设立分支机构。深化价格领域改革，防范物价过快上涨。完善国有资产管理体制。健全土地、资本、人力资源、技术等要素市场。

积极推进农村综合改革。坚持和完善农村基本经营制度，推进农村土地承包经营权有序流转。全面完成集体林权制度改革。深化征地制度改革，加快农村土地登记发证工作，逐步推进以证管地。

（四）着力抓好“双转移”和“双到”工作，促进区域协调发展

推动产业园区提升发展。发挥佛山（清远）省级示范性产业园的品牌优势，辐射带动各地产业园加快发展。支持佛山禅城（清新）和顺德（英德）产业转移园、民族工业园、华侨工业园建设，抓好特色产业承接。全力推进顺德（英德）区域合作产业园加快建设，努力打造成为全省区域经济创新合作发展试验示范区。坚持主导产业和园区招商模式，力促产业集聚发展有新局面，产业区域科学合理布局形成新格局。

提高招商引资质量。借助省政府组织的大型高层招商活动，围绕延伸产业链条，提升产业层次，加强对发达国家、跨国公司、龙头企业的招商推介，力争在引进中央企业、世界500强企业和实力民企上有新突破，使一批带动力强的优质项目落户清远，力保工业发展的良好势头和招商引资“热土”的优势。坚持引进项目与引进技术、管理、人才相结合，鼓励外资更多投向高新技术产业、现代服务业、清洁能源和节能环保等产业，提高利用外资水平。

提升城镇化水平。全面实施主体功能区规划试点工作，完善政策配套体系。加快推进一批“三旧”改造示范工程，促进城市规模扩张和功能、空间布局优化。加快中心城区第二水厂建设，进一步完善市政道路及中心城区、县城和中心镇供电、供水、污水处理等城市配套功能。以县为单位每年整治10%的自然村，在各县（市、区）集中力量打造1-2个名镇、名村，示范带动宜居城乡建设。建立健全农村劳动力转移就业服务体系，组织培训农村劳动力3.7万人，转移劳动力就业9万人。以建立人口合理流动机制为目的，加快户籍制度改革，推动农村人口向城镇集聚。进一步简化入户手续，抓好农民工积分制入户城镇工作。

基本完成扶贫开发任务。突出抓好产业扶贫，完善贫困户与龙头企业的互助联结机制，以产业化带动贫困农民脱贫。进一步完善协调机制，大力实施“订单、定向、储备”培训模式和“一户一技能”培训计划，通过转移就业帮助贫困群众脱贫。引导和扶持不具备生产生活条件的贫困村通过搬迁实现脱贫。加快出台配套政策，建立移民后续帮扶长效机制，妥善解决移民生产就业问题，确保“搬得出、稳得住”。积极推进互助金和小额贷款工作，实行帮扶工作动态管理。加强基层组织建设，充分发挥镇、村一级在推动扶贫开发中的主导作用。大力整合帮扶资金，广泛动员社会力量参与扶贫，确保提前基本完成扶贫开发任务。

进一步加快民族地区发展。着重解决民族地区发展遇到的问题，落实民族地区发展专项资金，加大民族地区交通、能源等基础设施建设力度，夯实经济发展基础。实施项目倾斜政策，优先安排民族地区农业、旅游和特色产业项目，加快民族地区农副产品加工、医药、生态旅游等产业发展。进一步加大少数民族地区教育、文化、卫生等事业的扶持力度。

（五）着力发展现代农业，提高新农村建设水平

发展壮大特色农业。加大农业结构调整力度，继续抓好家禽、水果、蔬菜等优势农产品基地建设，壮大特色产业带和特色基地规模，做大做强特色农业，不断提升清远特色农产品的知名度和市场占有率。认真落实各项强农惠农政策，提高粮食综合生产能力，稳定粮食生产。

大力发展现代农业。推进土地集中集约经营，积极引进和培育农业龙头企业，鼓励支持龙头企业发展农产品精深加工。加强农产品品牌建设，大力推进标准化生产。加快现代化大型农产品批发交易市场、集散中心和物流园区建设，加快组建一批大型农产品和农资购销流通企业集团。发挥供销合作社流通网络优势，加快新农村现代流通网络建设。鼓励农业企业与国内外大型农业集团、跨国公司开展合作。积极推进“食品农产品质量安全示范区”建设，加快农产品出口基地建设，扩大农产品出口规模。

加强农村基础设施建设。以民生水利建设为重点，全面完成城乡水利防灾减灾工程、病险水库除险加固任务。加快现代标准农田建设和中低产田改造，加强农业装备建设。积极推进农村电网改造，加快农村道路硬底化，提高农村公路的通达深度和畅通水平。实施农村危房改造工程，加快村庄整治。积极发展农村社会事业，提高农民生活质量。

（六）着力抓好节能减排节地工作，推动经济社会可持续发展

加大节能降耗工作力度。完善节能目标年度考核机制，加强重点耗能企业监管，全力落实节能措施。实施固定资产投资项目节能评估和审核制度，积极推广和使用新能源、新技术，大力推进清洁生产和资源综合利用，推进工业、交通、建筑、商贸酒店、公共机构等重点领域节能。严格节能准入门槛，建立健全落后产能退出机制。

狠抓污染治理和减排。严格执行主要污染物排放总量控制和前置审核制度。加强饮用水源水质保护，狠抓重点流域、区域、行业污染治理，加快污水处理设施建设，加大农村及城乡结合部环境整治力度。严格污染物排放标准，提高环保准入门槛。强化环境监测和执法监督，严肃查处环境污染事故。

加强生态建设。抓好城市绿化工程建设，改造提升绿化水平，继续开展生态市创建活动。认真抓好封山育林，继续推进生态建设工程、雨雪冰冻灾害森林生态修复工程、“市肺”工程建设。加强地质灾害防治，加快阳山国家地质公园建设。严格林地林木资源保护管理。

扎实推进节约集约用地。完善耕地保护考核机制，严守耕地保护红线，继续推进开发补充耕地工作。建立健全节约集约用地导向机制，引导建设用地向产业升级和城乡布局优化倾斜。继续推进城乡建设用地增减挂钩试点工作。加大违法用地整治，推进土地科学、高效使用。

（七）着力加强和改善民生，推进幸福清远建设

进一步稳定和扩大就业。推进公共就业服务向基层延伸，建立覆盖城乡常住人口的服务体系。多渠道开发就业岗位，大力推进创业带动就业，积极提供创业孵化服务，鼓励高校毕业生等青年自主创业，力争城镇新增就业5万人，下岗失业人员再就业1.5万人，创业带动就业2万人。实施技工教育倍增计划，开展适应产业调整升级的职业培训。

健全城乡社会保障和救助体系。抓好城镇职工和城镇居民基本医疗保险、新型农村合作医疗扩面工作。推进普通门诊医疗费用统筹，做好医疗保险关系转移接续和异地就医结算服务。着力抓好未参保人员养老保险扩面工作，大力推进新型农村社会养老保险。加快完善优抚安置保障体系和社会救助体系，加大城乡社会福利设施建设力度。落实孤儿最低养育标准。加强法律援助，保障困难群众合法权益。

大力推进文化强市建设。实施文化强市规划纲要，加快重点公共文化基础设施建设，改造、新建一批县城文化馆、图书馆、博物馆和乡镇综合文化站、社区文化室。大力实施广播电视村村通工程、全国文化信息资源共享工程、农家书屋建设、农村电影放映工程等文化惠民工程。落实基层文化工作者待遇，逐步加大财政对基层文化工作的扶持力度。充分挖掘历史文化、民族文化、福地文化、英石文化等文化资源内涵，大力发展文化生态旅游业。加快文化产业园建设，推动文化产业发展。办好首届幸福清远群众艺术花会。

加快教育、卫生等社会事业发展。抓好教育中长期改革和发展规划纲要的实施，全面启动创建省教育强市和普及学前教育工作。继续加强义务教育规范化学校建设，加快推进现代职业教育体系改革试验区建设。提高高中阶段教育普及水平。继续抓好市特殊学校、市公共实训中心和新职校建设。积极稳妥推进公立医院改革试点，全面完成医疗卫生体制五项重点改革。完善市急救网络体系，加快改造县级医院、乡镇卫生院、社区卫生服务中心（站），积极推进医疗卫生信息平台建设。深入开展全民健身运动，推进乡镇农民体育健身工程建设。统筹解决人口问题，促进人口长期均衡发展。落实计划生育基本国策，稳定低生育水平。按甲级标准重建市计划生育服务中心，改造升级人口计生信息系统，建立基于全员人口管理的动态个案数据库。积极推进全国文明城市创建工作。进一步推进殡葬管理工作。落实妇女儿童发展规划，切实维护妇女儿童合法权益。加大民族地区扶持力度，依法管理宗教事务，维护民族团结和宗教和谐。加强国防教育，深化国防动员指挥机构改革，提高国防动员和后备力量建设质量。切实做好双拥和优抚安置工作。继续做好残疾人、侨务、人防、气象、统计、档案、方志、红十字、对台等工作。

加强和创新社会管理。引导企业改善劳动条件，构建和谐劳动关系。切实抓好安全生产，防止重特大安全事故发生。继续实行领导干部联合接访和下访制度，依托县镇村三级综治信访维稳工作平台，加大社会矛盾纠纷排查和调解力度。加大食品药品监管力度，推进药品安全示范县（区）创建工作。建立和完善价格调节基金机制，提高市县两级政府市场基本生活物价调控统筹能力。严厉打击制假售假、走私行为，规范市场秩序。完善突发公共事件应急处置机制，提高预防和处置能力。深入开展法制宣传，扎实推进“六五”普法工作。狠抓社会治安综合治理，严厉打击各类刑事犯罪，建设平安清远。

在这里，我代表市人民政府承诺，今年集中力量为人民群众办好十件实事：一是建立低收入群众临时价格补贴与价格上涨联动机制，适时向优抚、低保和农村五保供养对象发放价格补贴。二是将家庭人均年收入低于1500元的困难群众全部纳入最低生活保障范围，县级福利院全部建成。三是确保省定67条贫困村集体经济收入达到3万元以上，7.6万贫困人口脱贫。四是完善高中阶段教育学校国家资助政策体系，中职学校免学费政策扩大至城市困难家庭学生。五是100%的政府办基层医疗卫生机构实施基本药物制度，动工建设市人民医院二期工程和市中医院综合大楼。六是启动市“四馆合一”、图书馆购书中心、全民健身广场工程，以满足人民群众日益增长的文化需求。七是继续实施城乡清洁工程，基本完成“三边”整治任务。八是加快推进清连二级公路大修工程，正式启动清三、清佛公路大修工程，完成500公里通自然村公路路面硬化工程，方便群众出行。九是解决2049户符合廉租住房和公共租赁住房保障条件的家庭住房问题。十是开展“食品安全年”活动，建设1个省级、10个市级食品安全示范店，每个乡镇建设1个县级食品安全示范店。

（八）着力加强自身建设，提高政府公信力和执行力

提高依法行政水平。认真落实人大及其常委会各项决议，依法向人大及其常委会报告工作。认真接受人民政协的民主监督，主动听取各民主党派、工商联、无党派人士和人民团体的意见建议。认真办理人大建议、政协提案。加强法治政府建设，规范行政执法，提高政府依法行政水平。进一步加大审计监督和财政监管力度，强化权力运行制约和资金使用监控，确保在土地出让、工程招投标、政府采购、资金拨付等方面严格依法办事、公开办事、按程序办事。

加大职能转变力度。强化社会管理和公共服务职能，把更多精力放在服务企业、监管市场和解决民生问题上。创新政务公开方式，主动接受公众和舆论监督，提高政务透明度。广泛听取群众和专家意见，促进民主、科学、依法决策。完善信用体系建设，加快构建信用政府。加强社会诚信宣传教育，营造诚实守信的社会环境。加强政风建设，进一步转变机关作风，继续精简压缩会议、文件。

进一步提高行政效率。创新政府管理方式，进一步推广电子政务和网上办事，提高办事效率。完善工作目标责任制，健全重点工作领导挂帅、整体推进制度。健全机关效能监察机制，严肃查处政令不畅、办事效率低下、自由裁量失当等问题。建立目标激励机制，强化重点工作、重点项目的协调与督查，确保工作落实。

狠抓廉政建设。推进政府机关和公务员队伍廉政建设，确保反腐倡廉各项措施落到实处。认真开展廉政风险防范工作，从最容易滋生腐败的环节入手，推进反腐倡廉制度建设，规范权力运行，坚持从源头上预防和治理腐败。切实纠正部门和行业不正之风，严肃查处损害企业和群众利益的行为。大力弘扬艰苦奋斗、勤俭节约的精神，倡导健康、文明、向上的生活方式。

各位代表，承继“十一五”辉煌，开创“十二五”伟业，是时代赋予我们的光荣使命。让我们更加紧密地团结在以胡锦涛同志为总书记的党中央周围，在市委的正确领导下，深入贯彻落实科学发展观，紧紧依靠全市人民，再接再厉，开拓创新，为实现“十二五”良好开局、加快幸福清远建设而努力奋斗！
